# Ultrasonic-Assisted Extraction and Structural Characterization of Chondroitin Sulfate Derived from Jumbo Squid Cartilage

**DOI:** 10.3390/foods10102363

**Published:** 2021-10-04

**Authors:** Kai-Ruei Yang, Ming-Fong Tsai, Chwen-Jen Shieh, Osamu Arakawa, Cheng-Di Dong, Chun-Yung Huang, Chia-Hung Kuo

**Affiliations:** 1 Department of Seafood Science, National Kaohsiung University of Science and Technology, Kaohsiung 811, Taiwan; karry0710karry@gmail.com (K.-R.Y.); l38982079@gmail.com (M.-F.T.); cyhuang@nkust.edu.tw (C.-Y.H.); 2 Biotechnology Center, National Chung Hsing University, Taichung 402, Taiwan; cjshieh@dragon.nchu.edu.tw; 3 Graduate School of Fisheries and Environmental Sciences, Nagasaki University, Nagasaki 852-8521, Japan; arakawa@nagasaki-u.ac.jp; 4 Department of Marine Environmental Engineering, National Kaohsiung University of Science and Technology, Kaohsiung 811, Taiwan; cddong@nkust.edu.tw

**Keywords:** jumbo squid (*Dosidicus gigas*) cartilage, chondroitin sulfate, ultrasonic-assisted extraction, mass transfer kinetics, response surface methodology, structural characterization, FTIR, NMR

## Abstract

Chondroitin sulfate (ChS) is usually used as an oral nutraceutical supplement, and has been popular in Asia, Europe, and United States for many years. In this study, a potential and sustainable source of ChS from jumbo squid (*Dosidicus gigas*) cartilage was explored; ultrasound-assisted extraction (UAE) was used to extract ChS from jumbo squid cartilage. The result of mass transfer coefficients based on Fick’s law showed that UAE had higher mass transfer efficacy. The response surface methodology (RSM) combined with Box–Behnken design (BBD) was employed to evaluate the effects of the extraction parameters. The optimal conditions were extraction temperature of 52 °C, extraction time of 46 min, and NaOH concentration of 4.15%. The crude extract was precipitated by 50% ethanol, which obtained a purified ChS with 23.7% yield and 82.3% purity. The purified ChS measured by energy-dispersive X-ray spectroscopy (EDX) had a carbon to sulfur molar ratio of approximately 14:1. The FTIR, ^1^H, and ^13^C NMR confirmed jumbo squid ChS were present in the form of chondroitin-4-sulfate and chondroitin-6-sulfate, with a 4S/6S ratio of 1.62. The results of this study provide an efficient process for production and purification of ChS, and are significant for the development and utilization of ChS from jumbo squid cartilage in the nutrient food or pharmaceutical industries.

## 1. Introduction

**Scheme 1:**
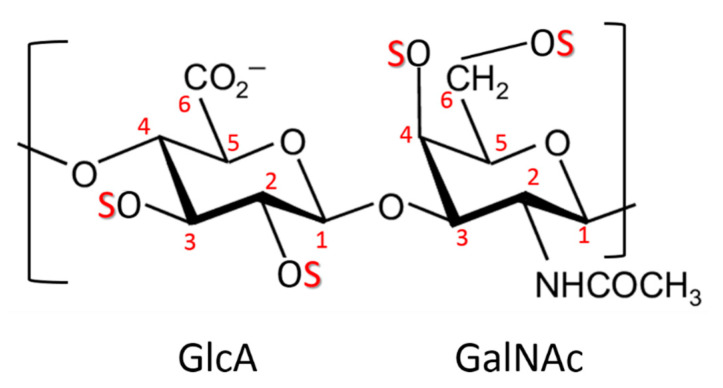
Structure of chondroitin sulfate. ChS A: GlcA-GalNAc(4S), ChS B: GlcA(2S)-GalNAc(4S), ChS C: GlcA-GalNAc(6S), ChS D: GlcA(2S)-GalNAc(6S), ChS E: GlcA-GalNAc(4, 6S).

Chondroitin sulfate (ChS) is an acid mucopolysaccharide, widely distributed in humans, other mammals, invertebrates, and some bacteria. ChS is a polymerized carbohydrate containing repeating disaccharide units of glucuronic acid (GlcA) and N-acetyl-galactosamine (GalNAc) connected by β-(1→3) glycosidic bonds and sulfated at different carbon positions [1,2], as depicted in Scheme 1. These repetitive disaccharides are usually monosulfated but, depending on the source, there may be disulfated disaccharides and trisulfated disaccharides in the main chain of the polysaccharide [3,4]. The sulfation positions of ChS can be divided into several patterns, such as ChS A, ChS B, ChS C, ChS D, and ChS E [5,6]. ChS is usually used as an oral nutraceutical supplement in the treatment of knee and hand osteoarthritis or joint pains, and has been popular in Asia, Europe, and United States for many years [7,8]. At present, ChS is mostly derived from animal sources, such as porcine, bovine and other mammals’ trachea and nasal septa [9], in addition to chicken keels [10]. However, due to the problems caused by bovine spongiform encephalopathy (BSE), H7N9 avian influenza, and other food chain crises, research on ChS from marine organism sources has attracted increasing attention [11,12]. At present, the main commerically available source of ChS used for health care is shark cartilage. The overexploitation of sharks has led to ecological risks derived from the reduction in biological resources.

Jumbo squid (*Dosidicus gigas*), distributed in the eastern Pacific Ocean from California to southern Chile, is an important economic fishery resource. The global capture production of jumbo squid was 74,7010 tons in 2016, according to the FAO report [13]. Commercially, this species has been caught to serve the European common market, Russia, China, Japan, Southeast Asia, and, increasingly, North and South American markets. In the processing of squid, more than 40% of the total weight is usually discarded as by-products, including viscera offal, skin, and cartilage [14]. The waste is mostly used for fish meal production or discarded. The disposal of this processing waste is becoming a major problem for industries, causing both environmental pollution and a loss of valuable nutrients. However, these wastes can be converted into high value-added products. For example, chitosan has been extracted from squid pen waste [15,16]. DHA and EPA enriched oil has been extracted from squid viscera [17,18]. Gelatin hydrolysates with antioxidant activity can also be obtained from jumbo squid gelatins [19]. However, jumbo squid cartilage, occupying about 2% of its body weight, is often discarded as waste during processing; therefore, it may be a potential source for ChS production.

The separation of ChS from cartilage usually includes the steps of hydrolysis of cartilage, breakdown of the proteoglycan core, protein elimination, and ChS recovery. The hydrolysis of cartilage is usually performed using alkali or enzyme methods. Alcalase has been used to hydrolyze the smooth hound cartilage at 50 °C for 24 h to obtain 2.5% ChS [20]. Although commercial papain has been used to degrade the proteoglycan of the cartilage of buffalo [21], lesser spotted dogfish [22], crocodile, and ray [23], at the temperature of 50–65 °C, enzymatic-assisted extraction requires a long time. Dilute alkali can break down the *O*-glycosidic bond between ChS and the core protein via β elimination reaction to release ChS. However, the excessive alkali concentration and high temperature causes the degradation of the released ChS [24]. To obtain the optimal extraction conditions, response surface methodology (RSM) is a practical statistical method that overcomes the shortcomings of the single-factor experiment method [25]. Simultaneously, it can be used to initiate a comprehensive statistical discussion on the influence of multiple factors, reflecting the importance of each factor, and the statistical results can be used to determine the optimal experimental conditions [26,27]. Recently, an ultrasonic-assisted extraction (UAE) technique has attracted increasing attention due to its inherent advantages, such as a reduction in extraction time, increase in extraction yield [28], enhanced mass transfer, and decrease in the thermal degradation of bioactive compounds [29,30]. UAE is mostly used in the extraction of bioactive ingredients from plants [31,32], and rarely applied to animal tissue. UAE has been used for extracting lipids from cobia liver [33] and Pacific white shrimp [34]. This is the first study focusing on using UAE to extract ChS from squid cartilage. Because the application of ChS or its derivatives depends on its quality and purity, it is highly important to use a high-yield extraction process to maintain the quality and purity of ChS. Therefore, the integration of UAE and RSM is beneficial because it may create a systematic, practical, and economical method for ChS extraction.

In this work, RSM and Box–Behnken designs were employed to investigate the effects of the extraction variables (extraction temperature, extraction time, and alkali concentration) and response (ChS concentration), in additio to obtain the optimal conditions for the extraction of ChS from jumbo squid. Mass transfer kinetics was employed to compare the efficiency of UAE and traditional extraction. The effects of ethanol concentration on the yield and purity of precipitated ChS were investigated. Finally, the structural characterization of purified ChS was analyzed by SEM, EDX, gel permeation chromatography, FTIR, and NMR.

## 2. Materials and Methods

### 2.1. Materials

The jumbo squid (*D. gigas*) was provided by Hsien Hua Frozen Foods Co., Ltd. (Kaohsiung, Taiwan). 1,9-Dimethylmethylene blue was purchased from Polysciences, Inc. (Philadelphia, PA, USA). Glycine and D-(+)- glucuronic acid were purchased from Sigma-Aldrich (St. Louis, MO, USA). Chondroitin sulphate sodium salt was provided by TCI (Tokyo, Japan). All other chemicals were analytical grade and are commercially available.

### 2.2. Pretreatment of the Jumbo Squid Cartilage

The cartilage was taken from the head of jumbo squid. After washing with water, the cartilage was homogenized by using an Osterizer Galaxie blender (Oster Corporation, Milwaukee, WI, USA). The resulting cartilage sludge was frozen and stored at −20 °C as raw material until use. The proximate composition of the cartilage sludge, as determined using AOAC methods [35], was 85.0 ± 0.1% moisture, 5.7 ± 0.1% carbohydrate, 5.5 ± 0.1% protein, 2.7 ± 0.3% fat, and 1.1 ± 0.1% ash.

### 2.3. Conventional Shaking Extraction

A quantity of 3 g of cartilage sludge was extracted with 3 mL of 3% NaOH aqueous solution in a 50 mL centrifuge tube. The centrifuge tubes were placed in an orbital shaking bath (100 rpm) at 50 °C for various extraction times. After extraction, the centrifuge tubes were taken from the orbital shaking bath, followed by centrifugation at 13,000 rpm for 10 min. The supernatant was used to analyze the content of ChS.

### 2.4. Ultrasonic-Assisted Extraction

A quantity of 3 g of cartilage sludge was extracted with 3 mL of 3% NaOH aqueous solution in a 50 mL centrifuge tube. The centrifuge tubes were placed in an ultrasonic bath (Elmasonic P 70 H, Elma, Siegen, Germany) and operated at 37 kHz with 100% output power for various extraction times. The extraction temperature was controlled at 50 ± 2 °C by adding ice to the ultrasonic bath. After extraction, the centrifuge tubes were taken from the ultrasonic bath, followed by centrifugation at 13,000 rpm for 10 min. The supernatant was used to analyze the content of ChS.

### 2.5. Determination of Mass Transfer Coefficients

Extraction is a mass transfer process; during the extraction, ChS is transferred from the cartilage to the liquid. The mass transfer rate is an important control factor for extraction efficiency. It is assumed that the diffusion of the solute in the solid is very rapid compared to the diffusion in the liquid. The mass transfer rate equation of the solute dissolved in the solution is as follows:

(1) $$\frac{N_{A}}{A}=k_{L}\left(C_{\mathrm{AS}}-C_{A}\right)$$

where N_A_ is the mass diffusion rate (mg ChS s^−1^), A is the surface area of cartilage sludge (m^2^), k_L_ is a mass-transfer coefficient (m s^−1^), C_AS_ is the saturation solubility of ChS in the solution (mg L^−1^), and C_A_ is the concentration of ChS in the solution at time t sec (mg L^−1^). 

Via the material balance in a batch system, the rate of accumulation of ChS in the solution is equal to Equation (1), shown as Equation (2):

(2) $$V\frac{{\mathrm{dC}}_{A}}{\mathrm{dt}}=N_{A}={\mathrm{Ak}}_{L}\left(C_{\mathrm{AS}}-C_{A}\right)$$

Integrating between t = 0 (C_A_ = 0) and t = t (C_A_ = C_A_) yields Equation (3):

(3) $$\ln(\frac{C_{\mathrm{AS}}}{C_{\mathrm{AS}}-C_{A}})=\frac{k_{L}A}{V}t$$

When the extraction volume and the concentration of cartilage sludge are fixed, A/V can be regarded as a constant to obtain Equation (4):

(4) $$\ln(\frac{C_{\mathrm{AS}}}{C_{\mathrm{AS}}-C_{A}})=k_{L}^{\prime }t$$

where k′_L_ is apparent mass transfer coefficient (S^−1^).

### 2.6. Experimental Design

The optimal conditions for extracting ChS from jumbo squid cartilage were determined using RSM. The Box–Behnken design with three levels and three factors was employed. The variables and levels selected were: NaOH concentration (2%, 4%, and 6%), extraction temperature (30, 40, and 50 °C), and extraction time (20, 40, and 60 min). Table 1 shows the levels of the independent factors and experimental designs as coded (0, 1, and −1) and uncoded (actual value). A total of 15 experimental runs, including different combinations of the three factors, were carried out in duplicate. SAS software (SAS Institute, Cary, NC, USA) was employed for the experimental design, data analysis, and model building. The experimental data were analyzed by response surface regression to fit the following second-order polynomial equation:

(5) $$Y=\beta_{k0}+\overset{3}{\underset{i=1}{\sum }}\beta_{\mathrm{ki}}X_{i}+\overset{3}{\underset{i=1}{\sum }}\beta_{\mathrm{kii}}X_{i}^{2}+\overset{2}{\underset{i=1}{\sum }}\overset{3}{\underset{j=i+1}{\sum }}\beta_{\mathrm{kij}}X_{i}X_{j}$$

where Y is the response (ChS concentration); β_k0_, β_ki_, β_kii_, and βk_ij_ are constant coefficients; and Xi and Xj are uncoded independent variables.

### 2.7. Purification of ChS by Ethanol Fractionation

The extracted ChS solution was adjusted to pH 7 by adding 3 M HCl; a certain amount of 95% ethanol was added to the solution so that the final ethanol concentration in the solution was 50, 60, 70, 75, and 80%, respectively. The precipitated ChS was centrifuged at 10,000 rpm for 20 min to remove the ethanol. The precipitated ChS was then re-dissolved with distilled water and freeze-dried. The ChS extraction yield (%) was expressed as: weight of freeze-dried ChS (g) per the weight of carbohydrates in the cartilage sludge × 100. The ChS purity was compared with the standard using the dimethylmethylene blue method.

### 2.8. Analysis

The ChS content was determined by the dimethylmethylene blue method [36]. The color reagent was prepared by dissolving 16 mg of dimethylmethylene blue in 1 L of water containing 3.04 g glycine, 2.37 g NaCl, and 95 mL 0.1 M HCl to obtain a solution with pH 3.0 and absorbance at 525 nm of 0.31. In the procedure, a 100 μL sample was placed in a test tube, 2.5 mL of dimethyl methylene blue reagent was added, the solution was shaken and mixed, and the absorbance was measured at 525 nm. Chondroitin sulphate sodium salt was used as a standard for the ChS content measurement. Protein concentration was estimated by the Bradford method using protein dye reagent concentrate (Bio-Rad, Hercules, CA, USA), and bovine serum albumin was used as the standard. The uronic acid content was measured by the colorimetric method [37], and D-glucouronic acid was used as the standard. The sample was hydrolyzed in 1 M HCl for 4 h before determining the sulfate content. The sulfate content was determined by the BaCl_2_-gelatin turbidity method [38], and K_2_SO_4_ was used as the standard. The molecular weight of the purified ChS was determined by an HPLC system, consisting of a Hitachi L-2130 HPLC pump and a Hitachi L-2490 refractive index detector (Hitachi, Tokyo, Japan) equipped with an 8.0 mm × 300 mm Shodex^TM^ SB-803HQ column (Shodex, Tokyo, Japan). Standard dextrans were used as molecular weight markers. The mobile phase was water at a flow rate of 1 mL min^−1^ and the injection volume was 20 µL of 0.5% sample.

### 2.9. SEM, FTIR and NMR Spectroscopy

A scanning electron microscope (SEM) and energy-dispersive X-ray spectroscopy (EDX) of purified ChS were performed using an environmental scanning electron microscope (FEI Quanta-200, Brno-Černovice, Czech Republic). Fourier-transform infrared spectroscopy (FTIR) was measured using a Horiba FT-730 spectrometer (Horiba Ltd., Kyoto, Japan). Dried ChS (2 mg) were mixed with KBr powder (100–200 mg) and pressed into thin discs using a hydraulic press. The spectra (4000–400 cm^−1^) were recorded with a resolution of 4 cm^−1^ and 64 scans were performed per sample. Nuclear magnetic resonance (NMR) spectroscopy was conducted on a Bruker AVANCE 600 MHz spectrometer to characterize the chemical structure of purified ChS. Prior to the NMR analysis, 10 mg of sample was dissolved in 1 mL D_2_O. The spectrometer frequency for NMR was 600 MHz.

## 3. Results

### 3.1. Comparison of Conventional Shaking Extraction and Ultrasonic-Assisted Extraction

**Figure 1:**
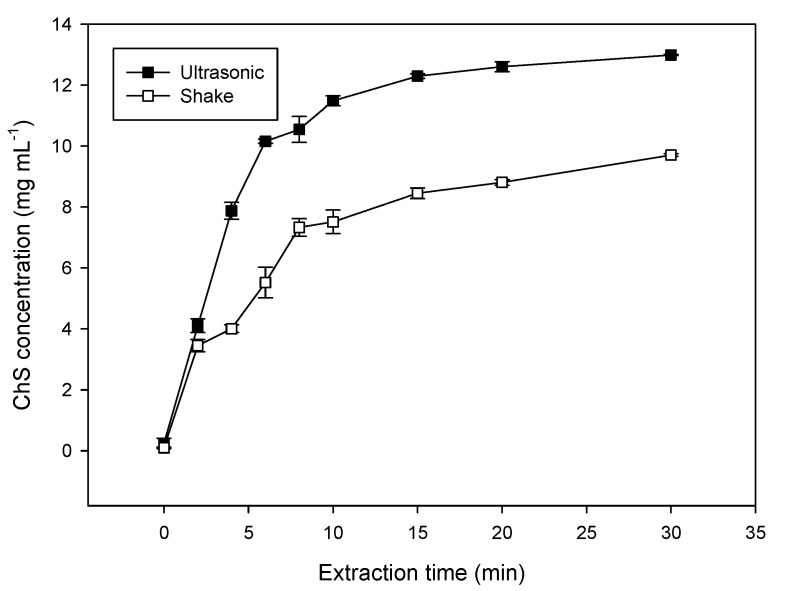
Comparison between conventional shaking extraction and UAE methods on the extraction of ChS. The UAE was performed at 37 kHz, 3% NaOH, and 50 °C, and the conventional shaking extraction was performed at 100 rpm, 3% NaOH, and 50 °C.

The conventional shaking extraction and UAE were evaluated for the extraction yield of ChS from jumbo squid cartilage. As shown in Figure 1, the ChS concentration of UAE was significantly greater than that of the conventional shaking extraction after extraction for 5 min. The ChS concentration of 12.5 mg mL^−1^ was achieved after ultrasonic extraction for 30 min, equivalent to a dissolution rate of 0.0069 mg mL^−1^ s^−1^. However, the conventional shaking extraction only obtained a ChS concentration of 9.7 mg mL^−1^, equivalent to a dissolution rate of 0.0054 mg mL^−1^ s^−1^. The result indicates that UAE increases the extraction yield 1.3-fold. The basic principle of UAE is the use of the strong cavitation, mechanical vibration, and heating effect of ultrasonic waves on the medium under certain conditions to enable the solvent to penetrate into the sample. In recent years, UAE has been applied to enhance the extraction polysaccharides from *Dictyophora indusiate* [39], green pea pods [40], marine algae [41], okra [42], pumpkin seeds [43], and straw mushroom [44]. The formation and rupture of bubbles by cavitation creates a shockwave to enhance the mass transfer and generate energy for β elimination reaction, thereby providing a beneficial effect on the release of ChS into the solution. Therefore, UAE can effectively increase the extraction yield and decrease the extraction time.

### 3.2. Mass Transfer Coefficients during the Extraction of ChS

**Figure 2:**
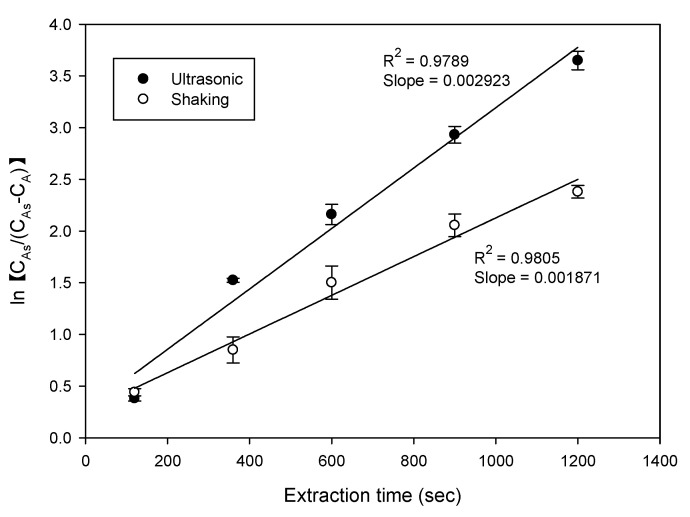
Determination of apparent mass transfer coefficients for extracting ChS during UAE and conventional shaking extraction. The UAE was performed at 37 kHz, 3% NaOH, and 50 °C, and the conventional shaking extraction was performed at 100 rpm, 3% NaOH, and 50 °C.

To explain the effects of UAE on the mass transfer enhancement, the mass transfer coefficients were calculated according to Fick’s law. Estimation of mass transfer coefficients is important for the determination of mass transfer rates, which can be calculated using the mass transfer rate equation by fitting the experimental data [29,45], as described in Section 2.5. The apparent mass transfer coefficient can be calculated from the plot of ln[C_AS_/(C_AS_ − C_A_)] versus extraction time, as shown in Figure 2. The determination coefficients of linear regression were 0.98 and 0.98 for conventional shaking extraction and UAE, respectively. Therefore, the slope of the linear regression can represent the apparent mass transfer coefficient (k’_L_) for the ChS extraction. The apparent extraction mass transfer coefficient of UAE (0.0029 s^−1^) was significantly higher than that of the conventional shaking extraction (0.0019 s^−1^), by 1.5-fold. The result indicates that UAE improved the mass transfer ability of molecules from solid phase to liquid phase, in addition to the extraction efficiency. This result can also be attributed to the assistance of the cavitation effect generated when the ultrasound acts on the extraction liquid [46,47]. Therefore, the UAE was used in the next stage for the optimization of the ChS extraction.

### 3.3. ChS Extraction Based on the Box-Behnken Design and RSM Model 

To evaluate the effect of extraction conditions (NaOH concentration, extraction time, and extraction temperature) on the extraction yield of ChS during UAE, a three-level and three-factor Box–Behnken design combined with the RSM for statistical analysis was employed in this study. The extraction conditions and experimental results are shown in Table 2. The manipulated factors and response values were analyzed to fit a regression equation that could predict the response value within the given range of the manipulated factors. The second-order polynomial equation of the RSM model is given for the extraction yields of ChS as below:

Y=3.04169266+0.9078031X_1_+0.1642753X_2_+0.074599709X_3_−0.099098X_1_^2^−0.01306X_2_X_1_+0.000090975254730963X_2_^2^ +0.01306X_3_X_1_−0.00094X_3_X_2_−0.00076X_3_^2^ (6)

The results of the ANOVA are shown in Table S1. The determination coefficient (R^2^ = 0.9955) with a small model *p*-value (*p* < 0.0001) indicates the acceptability of the model for estimating the predicted values from the regression equation. From Table S1, the linear terms of the three factors, three interaction terms, and two quadratic terms showed significant effects (*p* < 0.05), with the exception of the quadratic term of the extraction temperature (X_2_^2^), which did not show a significant effect (*p* > 0.05). Based on the results of ANOVA, all three factors were important factors highly correlated with the extraction yield of ChS.

### 3.4. Response Surface Analysis

**Figure 3:**
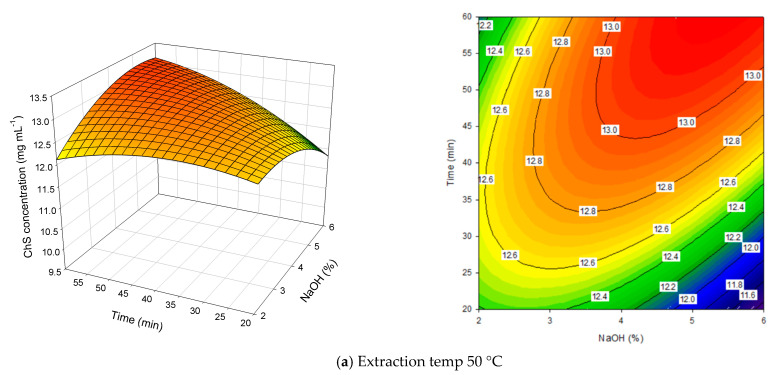

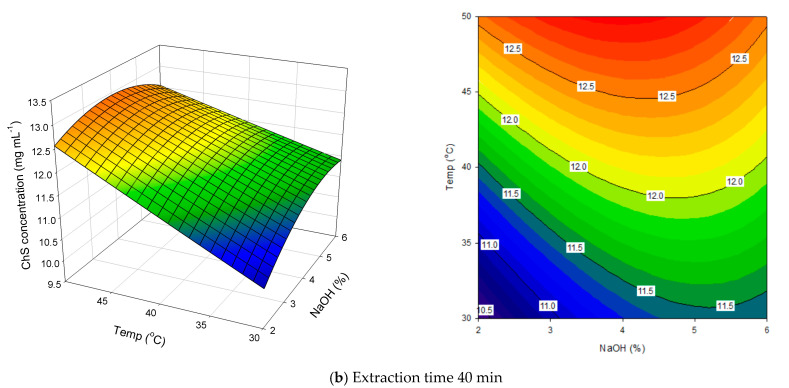
Response surface and contour plots show the effect of (**a**) NaOH concentration and extraction time, and (**b**) NaOH concentration and extraction temperature, on the extraction of ChS.

The response surface and contour plots can be obtained from the quadratic polynomial equation (Equation (6)) by fixing one of the factors to understand the relationships between the extraction factors and the response values. Figure 3a shows the response surface and contour plots of NaOH concentration and extraction time on the extraction of ChS. At the lowest extraction time (20 min) with the highest NaOH concentration (6%), the extraction of ChS was 11.3 mg mL^−1^. At the highest extraction time (60 min) with the NaOH concentration increasing from 2 to 5%, the extraction yield of ChS increased from 12.2 mg mL^−1^ to the highest peak of 13.0 mg mL^−1^. The extraction yield of ChS increased with the increase in extraction time and NaOH concentration. However, using higher NaOH concentration or extraction for a long time may cause the degradation of ChS and reduce the extraction yield. Figure 3b shows the effect of NaOH concentration and extraction temperature on the extraction of ChS. At the highest extraction temperature of 50 °C, the ChS concentration reached the highest peak of 12.9 mg mL^−1^ at the NaOH concentration of 4%, and showed a decreasing trend when the NaOH concentration was more than 4% because the ChS may degrade at a high NaOH concentration. In particular, the higher NaOH concentration was used; as a result, the yellower color of the extracted ChS solution was obtained.

### 3.5. Attaining Optimum Conditions

The optimum extraction conditions were determined by ridge analysis, which computed the estimated ridge of the maximum response for an increasing radius from the centre of the original design. The ChS concentration (estimated response; Y) at radius distances of 0, 0.6, 1.2, 1.8, 2.4, and 3.0 was calculated according to the RSM model (Equation (6)), as shown in Table 3. The actual experimental value of ChS concentration increased with the radius distance, reaching the maximum at a radius distance of 1.2. The ridge analysis showed that the optimal extraction condition was NaOH of 4.15%, extraction temperature of 52 °C, and extraction time of 46 min, which obtained a ChS concentration of 13.1 mg mL^−1^. When the radius distance was greater than 1.2, the actual experimental values of ChS concentration did not increase with the radius distance. These results may be due to the high temperature (>60 °C) causing the degradation of ChS under alkaline conditions. He et al. used high-intensity pulsed electric fields to extract ChS from fish bone, obtaining the maximum yield of 6.9 mg mL^−1^ at the NaOH concentration of 3.2% [24]. Zhao et al. reported that the optimal conditions for extraction of ChS from Chinese sturgeon cartilage were an NaOH to cartilage powder ratio of 9.2 and NaOH of 4.4%, but the extraction time was 3.9 h [48]. In comparison, the combination of UAE and RSM in this study can greatly reduce the extraction time and increase the extraction yield.

### 3.6. Purification of ChS by Ethanol Fractionation

The crude ChS solution extracted from the optimal extraction condition was purified by ethanol precipitation. ChS contains a large number of hydrophilic groups, such as carboxyl groups, hydroxyl groups, and sulfate groups. ChS is easily soluble in water but insoluble in organic solvents such as ethanol and acetone. The ethanol was added to the crude ChS solution and adjusted to different ethanol concentrations for ChS purification. The results of purified ChS from different ethanol concentrations are listed in Table 4. The yields of purified ChS obtained at 80, 75, 70, 60, and 50% ethanol concentrations were 37.8, 31.4, 28.1, 25.6, and 23.7% with the purities of 40.7, 64.6, 68.0, 70.8, and 82.3%, respectively. It can be seen that the yield decreased as the ethanol concentration decreased; however, the purity increased because the impurities such as proteins also precipitated at high ethanol concentration. However, ChS hardly precipitates at 40% ethanol concentration and is difficult to recover. Therefore, the highest purity (82.3%) with yield (23.7%) and lowest soluble protein (5.8%) were obtained at a 50% ethanol condition for the purification of ChS. Vazquez et al. reported that the highest purity of ChS was obtained by adding a ~1.1-fold volume of ethanol from *Prionace glauca* head wastes [49] and rabbit fish [50], which is consistent with our findings.

The repeating unit of ChS is glucuronic acid and N-acetyl-galactosamine connected by β-(1→3) glycosidic bonds; sulfation often occurs at the carbon position of the two sugar units. The contents of uronic acid and sulfate in the precipitated ChS are listed in Table 4. The contents of uronic acid and sulfate increased with decreasing ethanol concentration. The ChS obtained at 50% ethanol concentration showed a significantly higher content of uronic acid and sulfate (241.7 and 80.1 mg g^−1^, respectively) than those of the ChS obtained at 80% ethanol concentration (134.5 and 49.3 mg g^−1^, respectively). The content of uronic acid and sulfate is proportional to the purity of ChS, as shown in Table 4. The ChS isolated from smooth hound cartilage has 80.7% uronic acid and 21.5% sulfate [20]. The ChS isolated from sea cucumbers contained 225 mg g^−1^ of uronic acid and 431 mg g^−1^ of sulfate [51]. The difference in the content of uronic acid and sulfate may be due to the different sources of ChS. Based on the results, the precipitated ChS at a 50% ethanol concentration had higher purity, and content of uronic acid and sulfate; thus, it was used for subsequent structural analysis.

### 3.7. Characterization of Purified ChS

**Figure 4:**
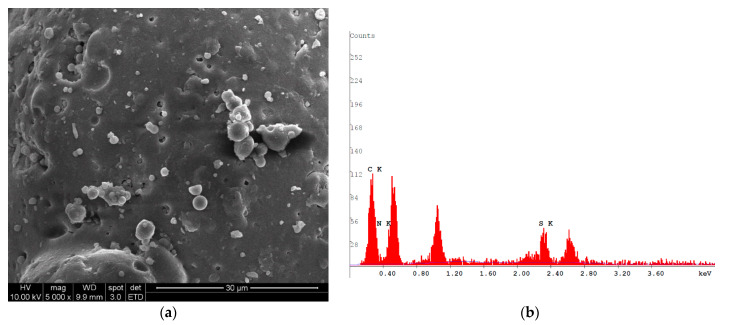
(**a**) SEM images (5000×) and (**b**) EDX spectra of purified ChS from jumbo squid cartilage.

**Figure 5:**
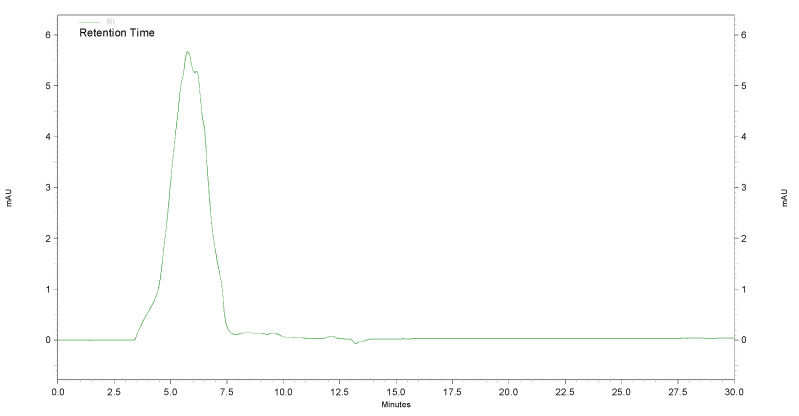
GPC profiles of purified ChS.

The morphology and elemental composition were measured using SEM/EDX. Figure 4a is the SEM image at 5000× magnification; the surface morphology of purified ChS is similar to that of typical ChS, as observed by Li et al. [52]. EDX analysis observed the element composition of ChS under the 5000× magnification of the SEM, as shown in Figure 4b. The ChS showed the weight percentages of carbon, nitrogen, and sulfur, of 74.1%, 12.0%, and 13.9%, respectively, which were converted into a mole ratio of ~14:2:1. Because a disaccharide unit of ChS has 14 carbon atoms, the results indicate that a disaccharide unit has one position for sulfation on average. The molecular weight distribution of the purified ChS was determined by gel permeation chromatography (GPC) using a Shodex^TM^ SB-803HQ column, with a calibrated curve as follows: Elution time = −1.4568 logMw +13.418. As shown in Figure 5, the peak was observed around 3∼7 min and the molecular weight was estimated by the elution time of the highest peak. GPC analysis showed that purified ChS had a single symmetrical peak with a molecular weight of 240 kDa. The molecular weight of ChS isolated from bovine nasal cartilage was 88 kDa [53]; from smooth hound cartilage, it was 69 kDa [20]; and from by-products of *Scyliorhinus canicula*, *Prionace glauca*, and *Raja clavate,* was between 43 and 60 kDa [54]. Rani et al. reported that the molecular mass of references chondroitin 4-sulphate, chondroitin 6-sulphate, and ChS from chicken keel bone were 70, 110, and 100 kDa, respectively [55]. The molecular weight of ChS from different sources differs. Compared with these reported studies, the purified ChS from jumbo squid had a higher molecular weight.

### 3.8. Structural Features of Purified ChS

**Figure 6:**
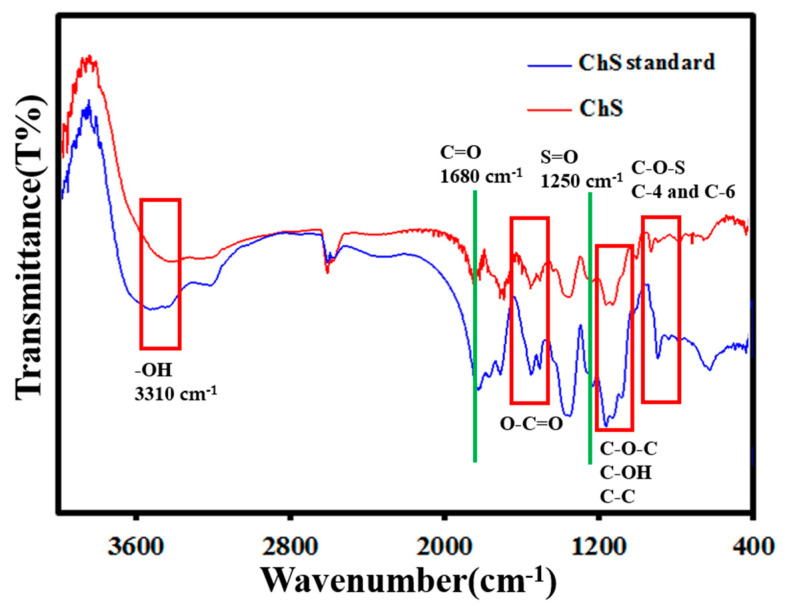
FTIR spectra of ChS standard (blue line) and purified jumbo squid ChS (red line) through 50% ethanol precipitation.

FTIR is a method commonly used to identify the functional groups of ChS. The FTIR spectrum of ChS standard (commercial ChS from shark cartilage, CAS 9082-07-9 belonging to ChS E) and purified ChS was measured from 4000 cm^−1^ to 400 cm^−1^ (Figure 6). The purified ChS result is consistent with the ChS standard results, and exhibits a broad band around 3310 cm^−1^ that comprises the -OH groups. The asymmetric stretch vibration of C=O of N-acetylgalactosamine (GalNAc) and glucuronic acid was visible at 1680 cm^−1^. The bending of O-C=O of uronic acids was evidenced by the two medium-size bands at 1379 and 1420 cm^−1^. The stretching band of S=O of sulfates was confirmed at 1250 cm^−1^. The absorbance bands at 1037, 1071, and 1135 cm^−1^ were ring vibrations of C-O-C, C-OH, and C-C, respectively, suggesting the occurrence of pyranose rings. The peak at approximately 857 cm^−1^ was used to identify chondroitin-4-sulfate, and the peak at 826 cm^−1^ was used to identify chondroitin-6-sulfate [23]. The C-O-S vibration of sulfate at C-4 and C-6 of GalNAc revealed the presence of peaks at 857 and 827 cm^−1^, respectively; this finding suggests that the hydroxy groups at C-4 and C-6 of GalNAc were substituted by sulfate groups for the ChS of jumbo squid.

**Figure 7:**
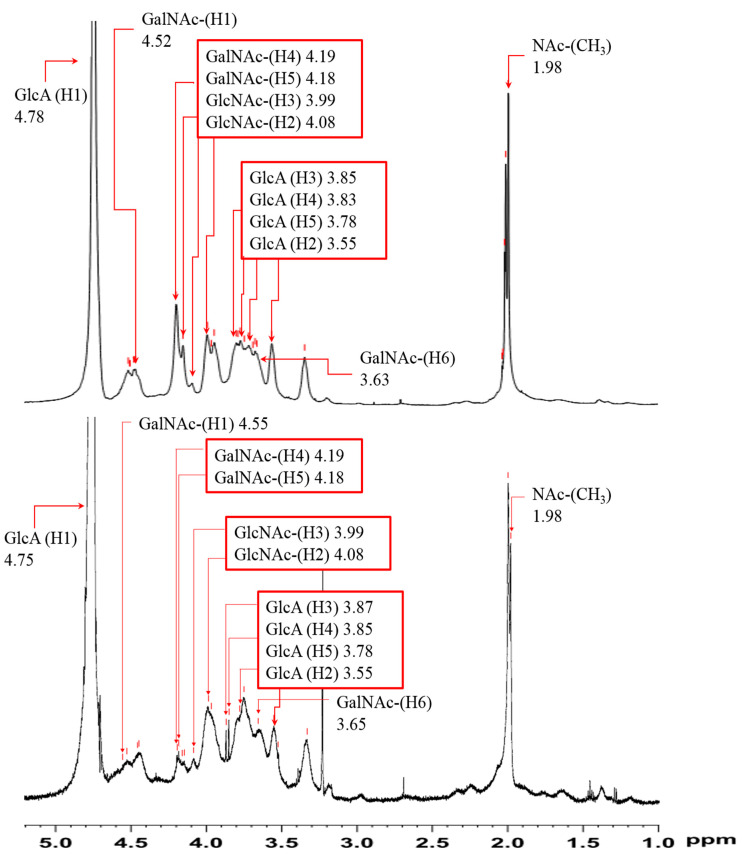
^1^H NMR spectrum of (**a**) ChS standard and (**b**) purified ChS measured at 600 MHz.

The ChS standard and purified ChS were characterized by ^1^H NMR spectra and are shown in Figure 7. The feature signals of purified ChS are listed in Table 5. The ChS signals at 4.75, 3.55, 3.87, 3.85, and 3.78 ppm were assigned to GlcA H1-H5, respectively, and the signals at 4.55, 4.08, 3.99, 4.19, 4.18, and 3.65 ppm were assigned to GalNAc H1-H6, respectively. In addition, the signal at 1.98 ppm indicated the methyl group of the n-acetyl-galactosamine structure. In contrast, similar results of NMR patterns have been shown in previous studies of ChS extracted from pig trachea, bovine trachea, and shark cartilage [56]. The proton signal of 4.55 and 3.65 ppm is H1 and H6, respectively, of the GalNAc sulfide substituted feature signal, indicating the disaccharide structure of GlcA-GalNAc-6SO_4_. In addition, the proton signals of 4.55, 4.19, and 3.65 ppm correspond to H1, H4, and H6, respectively, of disulfate substituted on the structure of the 4 and 6 position, indicating that the structures of chondroitin-4-sulfate and chondroitin-6-sulfate are disulfate substituted, and belong to chondroitin sulfated type E [57].

**Figure 8:**
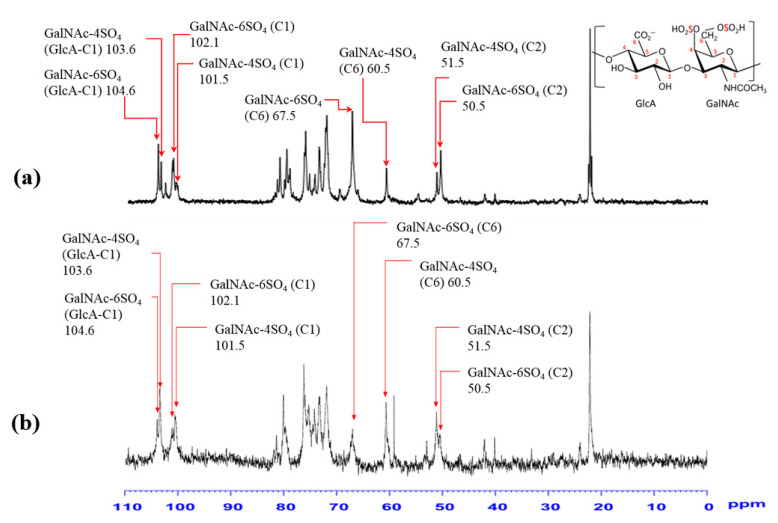
^13^C NMR spectrum of (**a**) ChS standard and (**b**) purified jumbo squid ChS measured at 600 MHz.

The ^13^C NMR spectra of ChS standard and purified ChS are shown in Figure 8. Both of the ^13^C NMR spectra were compared to whole signals around the 50–110 ppm region [48]. The signals at 104.6 and 103.6 ppm were assigned to GalNAc-6SO_4_ (GlcA-C1) and GalNAc-4SO_4_ (GlcA-C1), respectively. The signals at 102.1 and 101.5 ppm were assigned to C1 of GalNAc-6SO_4_ and GalNAc-4SO_4_, respectively. The C6 and C2 signals of GalNAc-6SO_4_ appeared at 67.5 and 50.5 ppm, respectively. The signals at 60.5 and 51.5 ppm were C6 and C2 of GalNAc-4SO_4_, respectively. Generally, the hydroxy groups of ChS at C-4 and C-6 of GalNAc, and the C-2 and C-3 of GlcA, are often substituted by sulfate groups. The classification and type of ChS depends on the sulfate group located at C-4 (ChS-A), C-6 (ChS-C), both C-4 and C-6 (ChS-E), C-6 of GalNAc and C-2 of GlcA (ChS-D), and C-4 of GalNAc and C-2 of GlcA (ChS-B) [5]. The jumbo squid ChS can be found at the sulfated positions at C-4 and C-6 of GalNAc, which can be classified as the E type. From the height of the peak, it can be seen that the proportion of GalNAc-4SO_4_ is greater than that of GalNAc-6SO_4_. The 4S/6S ratios calculated by the intensity of the NMR signal marked in Figure 8 were 0.51 and 1.62 for the ChS standard and purified ChS, respectively. It has been reported that the 4S/6S ratio of *Scyliorhinus canicula* was 0.59–0.63 [54]. Although the ChS from sharks and squids belongs to the ChS-E type, there is a significant difference in the 4S/6S ratio. In addition, the similar result via enzyme hydrolysis of the squid ChS procedure of disaccharide composition indicated the 4S/6S ratio is 1.41, and various sulfation degrees correspond to ChS-A (35.8%), ChS-C (12.6%), and ChS-E (44.5%) [58]. The NMR result is also consistent with the results observed by FTIR at wave numbers of 857 and 827 cm^−1^. In contrast, although sturgeon fish ChS showed a similar NMR pattern, the peak height of the GalNAc-6SO_4_ is greater than that of GalNAc-4SO_4_ [3]; this different proportion of sulfation may be due to the ChS from different species. Clearly, jumbo squid ChS has a higher 4S sulfation.

## 4. Conclusions

In this study, the UAE of ChS from jumbo squid was successfully developed using a Box–Behnken experimental design and RSM. The mass transfer coefficient results showed that the ultrasonic-assisted extraction had greater efficiency. The optimal UAE conditions were obtained by ridge-max analysis. At present, large-scale ultrasonic extraction equipment is commercially available, so this process has the potential for industrial production. The use of ethanol fractionation of crude ChS provided a means of low-cost production to obtain high purity ChS. The FTIR and NMR spectra showed that ChS was present in the form of chondroitin-4-sulfate and chondroitin-6-sulfate. In contrast to the common ChS from shark, which is currently commercially available, ChS from jumbo squid has higher 4S sulfation. To date, the ChS from marine sources has attracted increasing attention. The ChS from jumbo squid cartilage not only increases the value of waste, but also provides a cost-effective source of raw material.

## Figures and Tables

**Table 1:** Coding of experimental parameters and related levels.

Parameters	Symbol	Coded Levels		
		−1	0	1
NaOH (%)	X_1_	2	4	6
Temp (°C)	X_2_	30	40	50
Time (min)	X_3_	20	40	60

**Table 2:** Box–Behnken design and observed experimental data for UAE of ChS from jumbo squid cartilage.

Group	X_1_ NaOH (%)	X_2_ Temp (°C)	X_3_ Time (min)	Response ^a^ ChS (mg mL^−1^)
1	2 (−1) ^b^	40 (0)	20 (−1)	11.2 ± 0.3
2	2 (−1)	30 (−1)	40 (0)	10.4 ± 1.3
3	2 (−1)	50 (1)	40 (0)	12.5 ± 0.7
4	2 (−1)	40 (0)	60 (1)	11.3 ± 0.8
5	4 (0)	30 (−1)	20 (−1)	10.2 ± 0.6
6	4 (0)	50 (1)	20 (−1)	12.3 ± 0.1
7	4 (0)	40 (0)	40 (0)	12.2 ± 0.6
8	4 (0)	40 (0)	40 (0)	12.1 ± 0.9
9	4 (0)	40 (0)	40 (0)	12.1 ± 0.7
10	4 (0)	30 (−1)	60 (1)	11.7 ± 0.3
11	4 (0)	50 (1)	60 (1)	13.1 ± 0.4
12	6 (1)	40 (0)	20 (−1)	10.5 ± 0.5
13	6 (1)	30 (−1)	40 (0)	11.5 ± 0.9
14	6 (1)	50 (1)	40 (0)	12.6 ± 0.9
15	6 (1)	40 (0)	60 (1)	12.7 ± 0.5

^a^ Data are expressed as mean ± SD (n = 2); ^b^ The values −1, 0, and 1 are coded levels.

**Table 3:** Ridge analysis for the extraction of ChS.

Coded Radius	NaOH (%)	Temp (°C)	Time (min)	Estimated (mg mL^−1^)	Actual (mg mL^−1^)
0	4.00	40	40	12.1	11.8 ± 0.6
0.6	4.20	45	45	12.7	12.7 ± 0.9
1.2	4.15	52	46	13.2	13.1 ± 0.4
1.8	3.80	58	43	13.7	13.1 ± 0.3
2.4	3.36	64	39	14.2	13.0 ± 0.4
3.0	2.90	69	35	14.8	13.0 ± 0.2

**Table 4:** The effect of different ethanol concentrations on the ChS yield and its quality (purity and contents of soluble protein, uronic acid, and sulfate).

Ethanol (%)	Yield (%)	Purity (%)	Soluble protein (%)	Uronic Acid (mg g^−1^)	Sulfate (mg g^−1^)
80	37.8 ± 0.3 ^e^*	40.7 ± 1.8 ^a^	12.8 ± 0.1 ^bc^	134.5 ± 10.3 ^a^	49.3 ± 3.9 ^a^
75	31.4 ± 0.1 ^d^	64.6 ± 0.0 ^b^	12.9 ± 0.7 ^bc^	170.1 ± 13.4 ^b^	60.9 ± 3.3 ^b^
70	28.1 ± 0.5 ^c^	68.0 ± 1.9 ^b,c^	14.4 ± 0.9 ^c^	204.4 ± 3.2 ^c^	73.1 ± 1.0 ^c^
60	25.6 ± 0.1 ^b^	70.8 ± 4.9 ^c^	10.4 ± 0.1 ^b^	216.0 ± 5.3 ^cd^	75.0 ± 0.3 ^c^
50	23.7 ± 0.0 ^a^	82.3 ± 2.7 ^d^	5.8 ± 0.2 ^a^	241.7 ± 9.0 ^d^	80.1 ± 3.3 ^c^

* The different letters indicate a significant difference at *p* < 0.05.

**Table 5:** ^1^H NMR (600 MHz) chemical shift of purified jumbo squid ChS.

Residue	Proton	Current ^1^H Chemical Shift (ppm)
GlcA	H-1	4.75
	H-2	3.55
	H-3	3.87
	H-4	3.85
	H-5	3.78
GalNAc	H-1	4.55
	H-2	4.08
	H-3	3.99
	H-4	4.19
	H-5	4.18
	H-6	3.65
	NAc (CH3)	1.98

## Data Availability

Data is contained within the article.

## Supplementary Materials

The following are available online at https://www.mdpi.com/article/10.3390/foods10102363/s1, Table S1: ANOVA of response surface model of all independent variables.
