# Supplementary material for: Ultrasonic-Assisted Extraction and Structural Characterization of Chondroitin Sulfate Derived from Jumbo Squid Cartilage
Source: Foods. 2021 Oct 4;10(10):2363. doi: 10.3390/foods10102363 (PMC8535863; doi:10.3390/foods10102363)
Supplement: Supplementary file 1 [file foods-10-02363-s001.zip › foods-1386787-supplementary.pdf]

foods

Ultrasonic-Assisted Extraction and Structural Characterization of Chondroitin Sulfate Derived from Jumbo Squid Cartilage

Kai-Ruei Yang [1], Ming-Fong Tsai [1], Chwen-Jen Shieh [2], Osamu Arakawa [3], Cheng-Di Dong [4], Chun-Yung Huang [1] and Chia-Hung Kuo [1,*]

1 Department of Seafood Science, National Kaohsiung University of Science and Technology, Kaohsiung 811, Taiwan; karry0710karry@gmail.com (K.-R.Y.); l38982079@gmail.com (M.-F.T.); cyhuang@nkust.edu.tw (C.-Y.H.)
2 Biotechnology Center, National Chung Hsing University, Taichung 402, Taiwan; cjshieh@dragon.nchu.edu.tw
3 Graduate School of Fisheries Science and Environmental Studies, Nagasaki University, Nagasaki 852-8521, Japan; arakawa@nagasaki-u.ac.jp
4 Department of Marine Environmental Engineering, National Kaohsiung University of Science and Technology, Kaohsiung 811, Taiwan
* Correspondence: kuoch@nkust.edu.tw; Tel.: +886-7-361-7141 (ext. 23646)

Supplementary Materials:

Citation: Yang, K.-R.; Tsai, M.-F.; Shieh, C.-J.; Arakawa, O.; Dong, C.-D.; Huang, C.-Y.; Kuo, C.-H. Ultrasonic-assisted extraction and structural characterization of chondroitin sulfate derived from jumbo squid cartilage. *Foods* **2021**, *10*, x. https://doi.org/10.3390/xxxxx

Academic Editor: Firstname Lastname

Received: date
Accepted: date
Published: date

Publisher's Note: MDPI stays neutral with regard to jurisdictional claims in published maps and institutional affiliations.

Copyright: © 2021 by the authors. Submitted for possible open access publication under the terms and conditions of the Creative Commons Attribution (CC BY) license (https://creativecommons.org/licenses/by/4.0/).

Table S1. ANOVA of response surface model of all independent variables.

Source	Coefficient	Sum of Squares	d.f.	*F*-Value	*p*-Value
**Model**		10.9778	9	123.4175	< 0.0001**
Constant	3.0417		1		
**Liner**					
X_1-NaOH	0.9078	0.4827	1	48.8392	0.0009**
X_2-Temp	0.1643	5.4658	1	553.0435	< 0.0001**
X_3-Time	0.0746	2.6638	1	269.5324	< 0.0001**
**Interaction**					
X_1X_2	-0.0129	0.2649	1	26.8007	0.0035**
X_1X_3	0.0131	1.0918	1	110.4758	0.0001**
X_2X_3	-0.0009	0.1401	1	14.1756	0.0131*
**Quadratic**					
X_1^2	-0.0991	0.5802	1	58.7020	0.0006**
X_2^2	0.0001	0.0003	1	0.0309	0.8673
X_3^2	-0.0008	0.3386	1	34.2582	0.0021**
Residual		0.0494	5		
Lack of Fit		0.0434	3	4.8243	0.1765
Pure Error		0.0060	2		
R2	0.9955				

*Significant at $p \leq 0.05$. **Significant at $p \leq 0.01$.
